# Exploring BMI's mediating influence on cardiovascular risk correlations with the triglyceride-glucose index: using NHANES and CHARLS cohorts

**DOI:** 10.3389/fcvm.2025.1593413

**Published:** 2025-06-12

**Authors:** Yimeng Jia, Shuo Zhang, Junjie Liu

**Affiliations:** ^1^ College of Clinical Medicine, North China University of Science and Technology, Tangshan, China; ^2^ Department of Critical Care Medicine, Affiliated Hospital of North China University of Science and Technology, Tangshan, China

**Keywords:** cardiovascular disease, triglyceride-glucose index, body mass index, mediation analysis, NHANES, CHARLS

## Abstract

**Objective:**

This investigation employed population-based datasets to elucidate the pathophysiological interplay between triglyceride-glucose index (TyG) and incident cardiovascular disease (CVD), and quantify the extent to which body mass index (BMI) operates as a biological mediator within this association, utilizing a dual-cohort analytical framework.

**Methods:**

In this study, 17,976 Americans from the NHANES (1999–2020) and 6,218 Chinese from the CHARLS (2011–2020) were included. To investigate the intricate link between the TyG index, BMI and CVD, researchers employed weighted multiple logistic regression, linear regression, restricted cubic spline (RCS) analysis, mediation analysis, and subgroup analysis.

**Results:**

Among the study population, 1,895 Americans and 1,798 Chinese were diagnosed with cardiovascular disease (CVD). The regression analysis indicated that individuals in the higher quartile of the TyG index had a significantly greater risk of developing CVD (NHANES: *P* < 0.01, 95% CI: 1.11–1.78; CHARLS: *P* < 0.001, 95% CI: 1.37–1.89). In both surveys, participants with elevated TyG indices and BMI levels exhibited the highest incidence of CVD. The TyG index significantly affected CVD in both the NHANES and CHARLS cohorts. The total effect in the NHANES cohort was 1.438 × 10^−3^ (*P* < 0.001), and in the CHARLS cohort, it was 0.007 (*P* < 0.001).

**Conclusions:**

In this study, two independent cross-sectional cohort studies demonstrated significant positive correlations among TyG, BMI, and CVD. Multivariate analyses identified BMI as a partial mediator in the TyG-CVD pathway, with robust effect magnitudes remaining stable after controlling for age, sex, and other confounders.

## 1 Introduction

According to World Health Organization (WHO) surveillance data cardiovascular diseases (CVD) are the predominant contributor to global mortality, posing a considerable risk to human health. Heart disease involves structural and functional abnormalities, which include coronary artery disease, arrhythmias, congenital heart defects, valvular diseases, and heart failure (1). The high morbidity and mortality associated with heart disease not only affect individuals' quality of life but also impose substantial medical and economic burdens on society. According to WHO statistics, approximately 18 million people die from heart disease annually, with this number continuing to rise. While mortality rates remain high in aging high-income countries, the incidence of heart disease is also increasing sharply in developing nations due to shifting lifestyles and aging populations. Therefore, early and accurate prediction of heart disease, along with timely interventions, is crucial for reducing its incidence and mortality.

In recent years, research on cardiovascular disease pathogenesis has increasingly focused on metabolic factors. Diabetes, for instance, significantly burdens the heart, increasing the risk of heart disease (2). The TyG, determined from fasting glucose and triglyceride values, serves as a novel marker for insulin resistance and metabolic problems. Insulin resistance plays a critical role in the onset and progression of CVD, contributing to metabolic abnormalities that promote atherosclerosis and elevate heart disease risk (3–5). Prospective cohort studies suggest a strong association between TyG and cardiovascular outcomes, particularly coronary artery disease and myocardial infarction. Meta-analyses of population-based data reveal that elevated TyG levels independently predict higher incidence of ischemic heart events, with hazard ratios exhibiting statistical significance across diverse groups after multivariate adjustment for metabolic parameters and lifestyle covariates (6, 7).

Rosa Oh et al. demonstrated that elevated triglyceride-glucose index was a risk factor for cardiovascular events in adults with type 1 diabetes (8). Bingxue Wang et al. focused on the relationship between triglyceride-glucose roundness index and cardiovascular disease incidence in middle-aged and elderly Chinese (9). Chunxue Li et al. focused on the specific group of middle-aged and older women (10). Keke Dang et al. only one large survey was used in the investigation (11). The study used data obtained from NHANES (1999–2020) and CHARLS (2011–2020). NHANES provides extensive health and nutrition data, including dietary habits, lifestyle factors, and physiological indicators, while CHARLS offers detailed socio-economic, health, and retirement data for the Chinese population. The large and representative sample sizes from both databases allow for a comprehensive analysis of heart disease risk factors across diverse populations. By incorporating both NHANES and CHARLS, this study captures distinct demographic characteristics of Western and Eastern populations, enabling a more precise identification of common and population-specific risk factors for heart disease.

While extant literature has delineated the pathophysiological interplay between TyG index and cardiometabolic outcomes (11), the mediation pathways through anthropometric determinants remain underexplored. Capitalizing on harmonized data from NHANES and CHARLS, this investigation aims to examine the correlation between the TyG index and heart disease across these two large-scale datasets. Additionally, by employing multivariate-adjusted models, the study quantifies TyG's predictive accuracy for heart disease, identifying clinically relevant thresholds for risk stratification. Findings may inform novel insights for primary prevention strategies through improved biomarker utilization in cardiovascular risk assessment protocols.

## 2 Materials and methods

### 2.1 Study populations

NHANES, conducted biennially by the National Center for Health Statistics (NCHS), evaluated the health and nutritional status of 58,744 U.S. adults aged 20–85 years between 1999 and 2020. Participants were selected based on specific inclusion and exclusion criteria to ensure a representative sample of the population. Exclusions were made for: (i) missing Baseline data (age, sex, race, education, marital status, income, smoking situation) (*n* = 10,594); (ii) missing laboratory data (*n* = 29,601); (iii) weighted with zero weight (*n* = 330); (iv) missing BMI data and hypertension data (*n* = 243);

Information from CHARLS, covering the period from 2011 to 2020, was also incorporated into the analysis. Of the 25,762 baseline participants, exclusions were made for: (i) age < 45 years (*n* = 169); (ii) incomplete records for Baseline data (gender, age, marital status, education), LDL-C, or other CVD information in 2011 (*n* = 12,745); (iii) already had CVD in 2011 (*n* = 2,388); (iv) undetermined occurrence of CVD between 2011 and 2020 (*n* = 3,205); (v) missing BMI data (*n* = 1,037).

**Figure 1:**
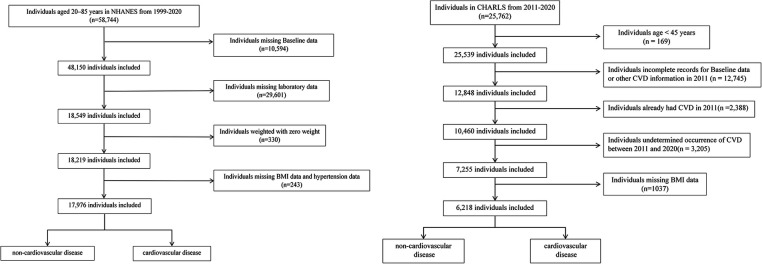
Flow chart of subject screening in NHANES and CHARLS.

Figure 1 outlines the step-by-step procedure, offering a clear visual guide to the methodology. After screening, the study incorporated 17,976 individuals from NHANES and 6,218 from CHARLS. Written informed consent was obtained from all participants, and both studies complied with the ethical standards of the 1975 Helsinki Declaration.

Diagnostic Criteria for CVD: CVD was identified through affirmative answers to two survey items: (i)A physician-confirmed diagnosis of heart disease (e.g., angina, myocardial infarction, heart failure, or related conditions); (ii)A physician-confirmed diagnosis of cerebrovascular accident (stroke).

### 2.2 Exposure and outcome variables

Fasting blood samples were collected and analyzed in a central laboratory. The TyG index, a marker of insulin resistance, was calculated as:

TyG = In[glucose [mg/dl] × TG [mg/dl]/2]; (ii) BMI= body mass (kg)/height^2^(m^2^) (12).

Cardiovascular disease (CVD), encompassing coronary heart disease, angina, myocardial infarction, congestive heart failure, or cerebrovascular events, served as the primary outcome, ascertained via self-reported clinician-confirmed diagnoses.

### 2.3 Data collection

Demographic information, such as sex, age, ethnicity, and education level; lifestyle factors, including smoking; medical history, for instance, hypertension; and biochemical indices, such as TC, TG, HDL, and LDL, were collected through standardized interviews and physical examinations. CVD risk served as the key outcome measure in this study, with participant eligibility evaluated via standardized diagnostic interviews incorporating two validated screening items. In NHANES, exposure, mediator, and outcome were measured at the same time point; in the CHARLS database, exposure, mediator were measured at the same time point. However, the CVD outcome occurred after the measurement of TyG and BMI.

### 2.4 Statistical analysis

Analytical weights were incorporated to address sampling complexity and enhance national generalizability. Continuous measures were expressed as mean ± standard deviation (SD), and categorical measures as frequencies (percentages). Comparative analyses utilized Student's *t*-test (normally distributed data), Mann–Whitney *U*-test (non-parametric), or *χ*² tests (categorical variables) based on distribution characteristics. The TyG index and BMI were stratified into quartiles (Q1–Q4) according to population distributions. Weighted multivariable regression models (logistic/linear) estimated adjusted odds ratios (ORs) and *β* coefficients with 95% CIs, controlling for demographics (age, sex) and clinical confounders. Sensitivity analyses further validated findings through subgroup stratification and alternative model specifications. Three multivariable regression frameworks were constructed: Model 1 (crude analysis), Model 2 (covariates included sociodemographic factors: age, sex, ethnicity, marital status, income-to-poverty ratio, educational attainment in NHANES; age, sex, marital, education in CHARLS), and Model 3 (additional adjustment for smoking status). Restricted cubic spline regression (RCS) revealed nonlinear dose-response patterns when *P* < 0.05. Stratified analyses were performed across gender, age groups (<60 vs. ≥60 years), and hypertension status, supplemented by interaction term assessments. All computations were executed in *R* 4.3.2 and SPSS 25.0, with statistical significance defined as two-tailed *P* < 0.05.

## 3 Results

### 3.1 Baseline characteristics

This cross-national analysis incorporated data from two population-based cohorts: 17,976 subjects from NHANES data set and 6,218 respondents from the CHARLS data set. Table 1 delineates the comparative demographic and clinical profiles of both study populations. Compared with individuals without cardiovascular diseases, those with cardiovascular diseases had a higher prevalence of hypertension (in NHANES, 75.38% vs. 33.63%; in CHARLS, 49.67% vs. 31.61%) and a higher smoking rate (in NHANES, 61.21% vs. 44.16%; in CHARLS, 37.93% vs. 37.49%). According to the distribution of the TyG index among patients, the TyG index was classified into four groups. In NHANES, the groups were Q1[, 8.185), Q2[8.185, 8.594), Q3[8.594, 9.026), and Q4 [9.03,). Correspondingly, in CHARLS, the groups were Q1 [6.606, 8.202), Q2 [8.202, 8.564), Q3 [8.564, 8.996), and Q4 [8.996, 11.986). Notably, across both population-based cohorts, participants exhibiting cardiovascular disease demonstrated significantly elevated concentrations of established metabolic risk markers—including triglycerides (TG) and fasting plasma glucose—relative to their non-cardiovascular diseases counterparts.

**Table 1:** Baseline characteristics of populations in NHANES and CHARLS.

Variable	NHANES			CHARLS		
	Non-cardiovascular disease (*n* = 16,081)	Cardiovascular disease (*n* = 1,895)	*P*-value	Non-cardiovascular disease (*n* = 4,420)	Cardiovascular disease (*n* = 1,798)	*P*-value
Age, years	45.32 ± 0.24	64.17 ± 0.46	<0.0001	57.30 ± 8.45	60.22 ± 8.85	<0.0001
poverty	3.03 ± 0.03	2.56 ± 0.06	<0.0001			
BMI, Kg/m^2^	28.51 ± 0.09	30.04 ± 0.24	<0.0001	23.28 ± 3.66	24.07 ± 4.00	<0.0001
Glucose, mg/dl	102.61 ± 0.31	116.58 ± 1.10	<0.0001	106.89 ± 28.71	111.91 ± 37.22	<0.0001
TG, mg/dl	121.87 ± 0.90	140.55 ± 2.34	<0.0001	125.18 ± 94.68	132.79 ± 87.07	<0.01
TC, mmol/L	5.08 ± 0.01	4.75 ± 0.04	<0.0001	5.00 ± 0.97	5.10 ± 0.98	<0.001
HDL, mmol/L	1.41 ± 0.01	1.33 ± 0.01	<0.0001	1.35 ± 0.39	1.31 ± 0.39	<0.001
LDL, mmol/L	3.03 ± 0.01	2.72 ± 0.03	<0.0001	3.02 ± 0.88	3.11 ± 0.91	<0.001
Non-HDL, mmol/L	3.67 ± 0.01	3.42 ± 0.04	<0.0001	3.65 ± 0.97	3.79 ± 0.99	<0.0001
TyG index	8.58 ± 0.01	8.84 ± 0.02	<0.0001	8.61 ± 0.63	8.73 ± 0.64	<0.0001
Sex, *n* (%)			<0.0001			0.04
Female	8,516 (52.58%)	816 (45.54%)		2,381 (53.87%)	1,022 (56.84%)	
Male	7,565 (47.42%)	1,079 (54.46%)		2,039 (46.13%)	776 (43.16%)	
Race, *n* (%)			<0.001			
white	7,379 (70.12%)	1,110 (76.00%)				
non-white	8,702 (29.88%)	785 (24.00%)				
Marital, *n* (%)			<0.0001			
Married	8,436 (54.08%)	1,023 (58.51%)				
Living with partner	1,263 (7.59%)	72 (4.39%)				
Separated	486 (2.12%)	58 (2.37%)				
Divorced	1,513 (9.83%)	244 (12.38%)				
Widowed	1,051 (4.79%)	361 (16.13%)				
Never married	3,332 (21.59%)	137 (6.23%)				
Education, *n* (%)			<0.0001			0.16
More than High School	8,459 (52.60%)	767 (40.47%)		49 (1.11%)	25 (1.39%)	
high school graduate	3,661 (22.77%)	463 (24.43%)		1,333 (30.16%)	494 (27.47%)	
Less than High School	3,961 (24.63%)	665 (35.09%)		3,038 (68.73%)	1,279 (71.13%)	
Smoke, *n* (%)			<0.0001			<0.0001
never	8,980 (55.84%)	735 (38.79%)		2,763 (62.51%)	1,116 (62.07%)	
Current/ former	7,101 (44.16%)	1,160 (61.21%)		1,657 (37.49%)	682 (37.93%)	
Hypertension, *n* (%)			<0.0001			<0.0001
no	10,191 (66.37%)	444 (26.42%)		3,023 (68.39%)	905 (50.33%)	
yes	5,890 (33.63%)	1,451 (73.58%)		1,397 (31.61%)	893 (49.67%)	
Diabetes, *n* (%)			<0.0001			<0.0001
no	13,696 (88.29%)	1,145 (65.36%)		3,871 (87.58%)	1,491 (82.93%)	
yes	2,385 (11.71%)	750 (34.64%)		549 (12.42%)	307 (17.07%)	

Data were expressed as mean ± SD for continuous variables and percentage for categorical variables. SD, standard deviation.

TG,triglycerides; TC, total cholesterol; HDL, high density lipoprotein cholesterol; LDL, low density lipoprotein cholesterol; Non-HDL-C, non-high-density lipoprotein cholesterol; TyG, triglyceride-glucose index.

### 3.2 Logistic regression and restricted cubic spline analysis

**Figure 2:**
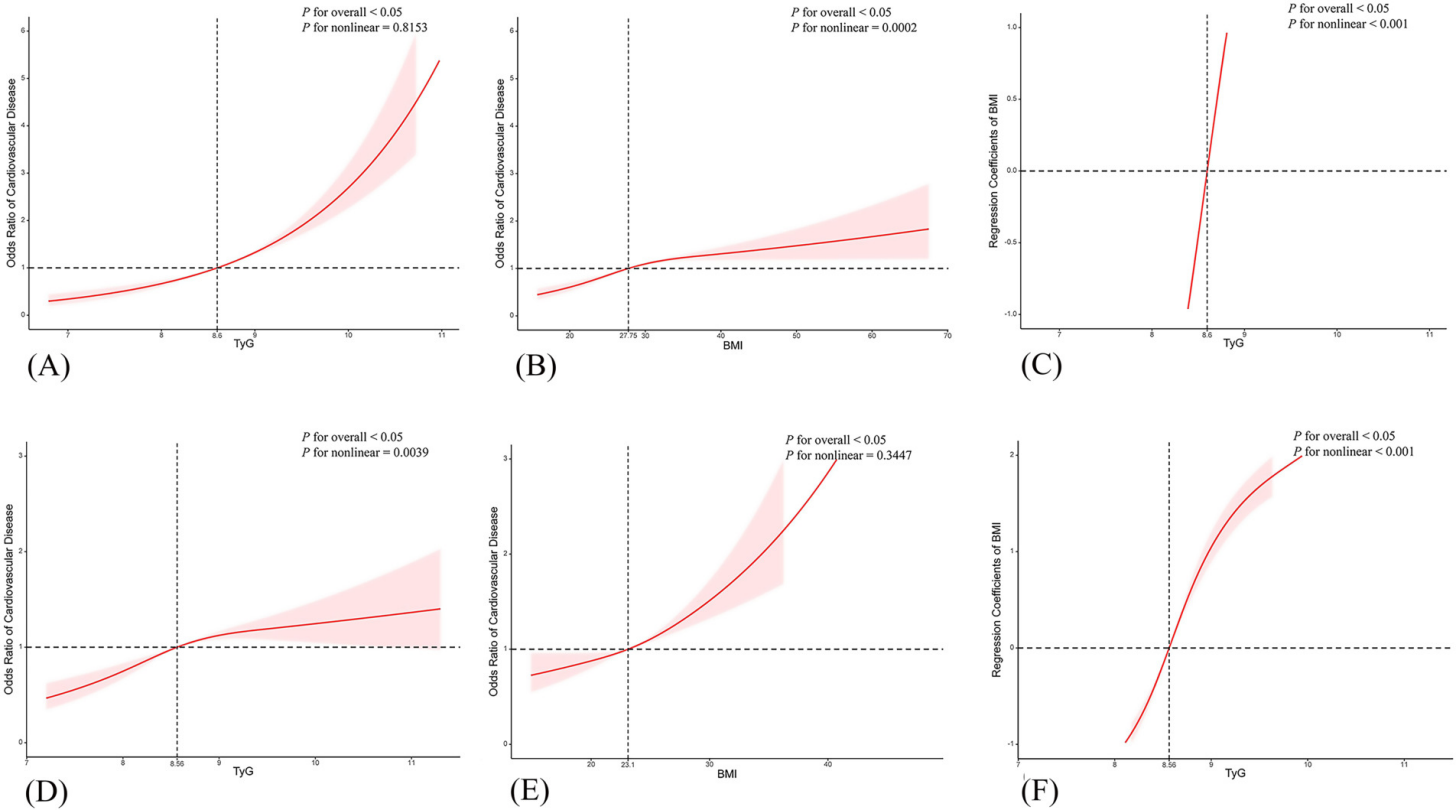
Restricted cubic spline (RCS) curves for the associations of TyG index, BMI, and increased CVD risk. Panels **(A–C)** show RCS curves from the NHANES data set (adjusted for age, sex, eth, marital, poverty, education, smoke), while panels **(D–F)** show RCS curves from the CHARLS data set (adjusted for age, sex, marital, education, smoke).

Table 2 delineated TyG index-CVD relationships across both cohorts. Specifically, NHANES data demonstrated elevated cardiovascular risk in the higher TyG quartiles (Q2–Q4) (*P* < 0.05). The TyG index values were 1.39 (95% CI: 1.12, 1.73) for Q2, 1.89 (95% CI: 1.53, 2.34) for Q3, and 2.90 (95% CI: 2.32, 3.62) for Q4 in the unadjusted model (Model 1). Following comprehensive adjustment in Model 3, the fourth TyG quartile (Q4) maintained statistical significance with an odds ratio of 1.41 (95% CI 1.11–1.78), accompanied by a significant exposure-response gradient across quartiles (*P*-trend = 0.03). In CHARLS, TyG quartiles demonstrated positive correlations with CVD risk, showing persistent trend significance (*P* < 0.001) across progressively adjusted models (Models 1–3). In unadjusted analyses, TyG Q4 demonstrated an OR of 1.61 (95% CI 1.37–1.88, *P* < 0.001). This association persisted through progressive adjustment models (Model 2: 1.61, 1.37–1.90; Model 3: 1.61, 1.37–1.89; *P* < 0.001 for all). When evaluating BMI as an independent exposure, each quartile elevation correlated with incremental CVD risk elevation. Post full adjustment (Model 3), BMI Q2–Q4 exhibited OR ranges of 1.11–2.06 (NHANES) and 1.14–2.08 (CHARLS), as detailed in Table 2. Following multivariate adjustment for demographic confounders (age, sex, race, marital status) and socioeconomic factors (poverty status, educational attainment), we employed restricted cubic spline (RCS) modeling to evaluate potential nonlinear associations between the TyG index and CVD risk. The results (Figure 2) indicated the NHANES cohort exhibited a statistically significant nonlinear relationship (*P* = 0.8153), with a threshold value of 8.6 for CVD risk. This analysis suggests a marked elevation in CVD risk when the TyG index exceeds 8.6, indicating that the TyG index may function as a continuous biomarker for cardiovascular risk stratification. In contrast, the CHARLS analysis demonstrated a statistically significant linear association (*P* = 0.0039), where exceeding the TyG threshold of 8.56 was associated with a substantial increase in CVD risk. When analyzing BMI as an independent predictor of CVD risk, RCS modeling revealed distinct patterns across cohorts: In the NHANES population, a significant nonlinear association emerged between BMI and CVD risk (*P* = 0.0002), with a critical threshold identified at 27.75 kg/m². This suggested abrupt risk escalation above this BMI level. Conversely, the CHARLS study demonstrated no statistically significant relationship (*P* = 0.3447), however, a linear trend was observed where CVD risk gradually increased with BMI exceeding 23.1 kg/m².

**Table 2:** Odds ratios of increased CVD risk by TyG index and BMI in the NHANES and the CHARLS.

Exposure variables	NHANES			CHARLS		
	OR (95% CI)	OR (95% CI)	OR (95% CI)	OR (95% CI)	OR (95% CI)	OR (95% CI)
	Model1	Model2	Model3	Model1	Model2	Model3
TyG index						
Q1	1.00 (Reference)	1.00 (Reference)	1.00 (Reference)	1.00 (Reference)	1.00 (Reference)	1.00 (Reference)
Q2	1.39 (1.12,1.73)**	0.87 (0.68,1.10)	0.87 (0.68,1.10)	1.25 (1.06,1.47)**	1.25 (1.06,1.48)**	1.26 (1.07,1.48)**
Q3	1.89 (1.53,2.34)***	1.03 (0.82,1.30)	1.02 (0.81,1.29)	1.53 (1.31,1.80)***	1.51 (1.28,1.77)***	1.5 (1.28,1.77)***
Q4	2.90 (2.32,3.62)***	1.44 (1.13,1.82)**	1.41 (1.11,1.78)**	1.61 (1.37,1.88)***	1.61 (1.37,1.90)***	1.61 (1.37,1.89)***
BMI						
Q1	1.00 (Reference)	1.00 (Reference)	1.00 (Reference)	1.00 (Reference)	1.00 (Reference)	1.00 (Reference)
Q2	1.34 (1.10,1.62)**	1.04 (0.84,1.29)	1.11 (0.90,1.38)	1.02 (0.86,1.19)	1.13 (0.95,1.33)	1.14 (0.96,1.35)
Q3	1.71 (1.40,2.10)***	1.33 (1.07,1.67)**	1.46 (1.17,1.82)***	1.15 (0.98,1.35)	1.31 (1.11,1.55)***	1.31 (1.11,1.54)***
Q4	1.94 (1.59,2.36)***	1.88 (1.51,2.34)***	2.06 (1.67,2.54)***	1.73 (1.49,2.02)***	2.07 (1.76,2.44)***	2.08(1.76,2.45)***

** *P* < 0.01.

*** *P* < 0.001.

NHANES: model 1: no adjusted; model 2: age, sex, eth, marital, poverty, education; model 3: age, sex, eth, marital, poverty, education, smoke; CHARLS: model 1: no adjusted; model 2: age, sex, marital, education; model 3:age, sex, marital, education, smoke.

### 3.3 Linear regression and subgroup analysis

Linear regression analysis demonstrated that within every model (Model 1–3), the TyG index in both surveys exhibited a significant association with BMI, with findings detailed in Table 3. When compared with Q1, the associations between TyG levels in Q2, Q3, and Q4 and BMI were statistically significant, and the effect sizes showed an increasing trend. These findings demonstrate a dose-dependent relationship between TyG index elevation and increased BMI magnitude, reinforcing the robustness of their positive correlation. Notably, multivariable adjustment for potential confounders maintained the strength of this association. Stratified analyses across demographic subgroups—including sex (male vs. female), age stratification (<60 vs. ≥60 years), and hypertension status—revealed no statistically significant interaction effects (*P* for interaction > 0.05), as detailed in Table 4.

**Table 3:** Linear regression analysis of the association between TyG and BMI in NHANES and CHARLS.

Exposure variables	NHANES			CHARLS		
	OR (95% CI)	OR (95% CI)	OR (95% CI)	OR (95% CI)	OR (95% CI)	OR (95% CI)
	Model1	Model2	Model3	Model1	Model2	Model3
TyG index						
Q1	1.00 (Reference)	1.00 (Reference)	1.00 (Reference)	1.00 (Reference)	1.00 (Reference)	1.00 (Reference)
Q2	1.96 (1.58,2.33)***	2.06 (1.67,2.44)***	2.12 (1.73, 2.50)***	0.86 (0.61,1.12)***	0.78 (0.53,1.03)***	0.78 (0.53,1.03)***
Q3	3.76 (3.37,4.16)***	3.9 (3.49, 4.31)***	3.96 (3.55, 4.37)***	1.65 (1.40,1.91)***	1.56 (1.31,1.81)***	1.56 (1.31, 1.80)***
Q4	5.42 (5.05,5.80)***	5.56 (5.15,5.97)***	5.63 (5.22, 6.05)***	2.9 (2.64,3.15)***	2.75 (2.50,3.00)***	2.73 (2.48, 2.97)***

*** *P* < 0.001.

NHANES: model 1: no adjusted; model 2: age, sex, eth, marital, poverty, education; model 3: age, sex, eth, marital, poverty, education, smoke; CHARLS: model 1: no adjusted; model 2: age, sex, marital, education; model 3:age, sex, marital, education, smoke.

**Table 4:** Subgroup analysis in NHANES and CHARLS.

Variables	NHANES			CHARLS		
	OR (95% CI)	*P*	P for interaction	OR (95% CI)	*P*	P for interaction
All patients						
Age			0.188			0.484
<60 years	1.397 (1.108,1.760)	0.005		1.289 (1.150, 1.444)	<0.0001	
≥60 years	1.186 (1.017,1.382)	0.029		1.377 (1.201,1.581)	<0.0001	
Gender			0.17			0.772
Male	1.338 (1.120,1.598)	0.002		1.354 (1.192, 1.537)	<0.0001	
Female	1.105 (0.897,1.362)	0.344		1.303 (1.154,1.470)	<0.0001	
Hypertension			0.62			0.701
No	1.174 (1.002,1.376)	0.047		1.247 (1.103,1.409)	<0.001	
Yes	1.234 (0.939,1.622)	0.131		1.187 (1.043,1.351)	0.010	

### 3.4 Mediation analysis

**Figure 3:**
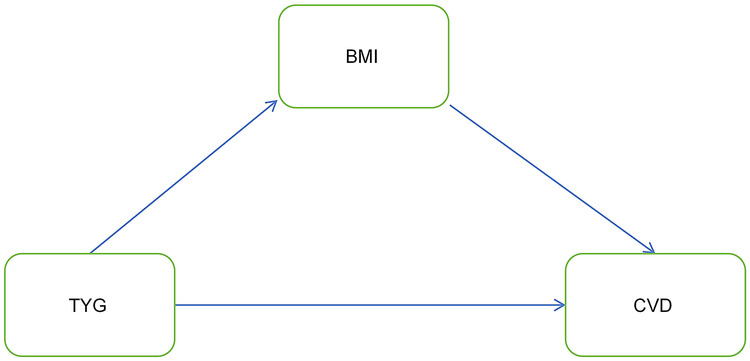
Mediation effect model diagram.

Currently, the degree to which the TyG index mediates cardiovascular disease occurrence via BMI remains unclear. This motivates us to investigate the mediating role of BMI in the association between them. Figure 3 illustrates the association model among the three. In both the NHANES and CHARLS cohorts, the total effect of the TyG index on CVD was significant, as shown in Table 5.

**Table 5:** Mediation analysis of TyG and CVD risk in the NHANES and CHARLS.

Effect	NHANES			CHARLS		
	Estimate (95% CI)	Estimate (95% CI)	Estimate (95% CI)	Estimate (95% CI)	Estimate (95% CI)	Estimate (95% CI)
	Model1	Model2	Model3	Model1	Model2	Model3
Total Effect	0.0000188 (0.000018001,0)***	0.002209 (0.002131,0)***	0.002335 (0.002243,0)***	0.011 (0.008,0.014)***	0.011 (0.007,0.014)***	0.011 (0.007,0.014)***
ACME (average)	0.000001594 (0.000001540,0)***	0.000692 (0.000675,0)***	0.000897 (0.000873,0)***	0.003 (0.001,0.01)***	0.003 (0.002,0.008)***	0.003 (0.002,0.008)***
ADE (average)	0.000017206 (0.000016455,0)***	0.001517 (0.001448,0)***	0.001438 (0.001361,0)***	0.008 (0.006,0.01)***	0.007 (0.005,0.008)***	0.007 (0.005,0.008)***

*** *P* < 0.001.

NHANES: model 1: no adjusted; model 2: age, sex, eth, marital, poverty, education; model 3: age, sex, eth, marital, poverty, education, smoke; CHARLS: model 1: no adjusted; model 2: age, sex, marital, education; model 3:age, sex, marital, education, smoke.

In the NHANES cohort, in the fully adjusted Model 3, the total effect (*β* = 2.355 × 10^−^³ *P* < 0.001) was decomposed into an indirect mediating effect via BMI (*β* = 8.97 × 10^−4^, *P* < 0.001) and a strong direct effect (*β* = 1.438 × 10^−3^, *P* < 0.001), indicating partial mediation. The proportion mediated (PM) through the pathway was 38.4%, indicating partial mediation of the effect and suggesting BMI exerts both metabolic pathway influence and direct physiological effects. In the CHARLS cohort, in the fully adjusted Model 3, the total effect *β* = 0.011, *P* < 0.001) had a modest but significant indirect effect (*β* = 0.003, *P* < 0.001) and a predominant direct effect (*β* = 0.007, *P* < 0.001). Pathway-specific mediation contributed 31.3% to the overall effect magnitude (PM = 31.3%).

## 4 Discussion

This investigation utilized pooled data from two multinational cohorts to investigate the dose-response association between triglyceride-glucose (TyG) index and incident cardiovascular disease (CVD). As a pathology persisting as a preeminent contributor to global mortality, CVD imposes substantial public health burden with profound socioeconomic repercussions across aging populations (13). Emerging studies have underscored the pivotal contribution of insulin resistance to the pathophysiological mechanisms underlying cardiovascular disorders (14), and the TyG index, serving as a surrogate indicator of insulin resistance and metabolic dysregulation, has emerged as a promising candidate biomarker for early-stage cardiovascular risk stratification in clinical and epidemiological settings (15).

Mechanistically, TyG index-measured insulin resistance induces elevated plasma free fatty acids, stimulating hepatic triglyceride/apolipoprotein B-containing LDL synthesis while reducing HDL clearance—thereby accelerating atherogenesis. This aligns with epidemiological evidence linking TyG-indexed metabolic dysregulation to coronary event risk, maintaining predictive value beyond conventional factors (16). Prospective cohort investigations have substantiated robust correlations between the TyG index and adverse cardiometabolic outcomes, encompassing acute coronary syndromes, cerebrovascular accidents, cardiovascular mortality, and diabetes mellitus progression (17–19). The TyG index's association with cardiovascular disease risk is stronger than that of glucose or triglyceride levels alone (20).

Emerging evidence indicates that elevated TyG index levels are associated with heightened coronary revascularization risk in normoglycemic populations. Notably, this biomarker demonstrates particular prognostic utility for ACS patients without diabetes who exhibit suboptimal LDL-C control (<1.8 mmol/L) (21). Prospective studies have also reported that in non-diabetic individuals, the incidence of CVD increases across TyG index quartiles, with hazard ratios for CVD (1.484), stroke (1.687), and myocardial infarction (1.402) in the highest TyG quartile compared to the lowest (22).

Mediation analysis further revealed that BMI partially mediated the relationship between the TyG index and CVD risk. Traditionally, obesity has been considered as an independent predictor of various cardiovascular diseases, including dyslipidemia, type 2 diabetes, hypertension, and sleep disorders (23). Adipose tissue functions not merely as a lipid storage depot but also as a critical endocrine organ. This endocrine organ modulates systemic energy balance and insulin signaling through adipokine secretion. Obesity-induced alterations in adipokine profiles contribute to insulin resistance and metabolic dysregulation (24). Emerging evidence highlights obesity as a critical driver of insulin resistance. Leveraging data from two longitudinal population-based studies, our analyses revealed that BMI significantly mediates the association between TyG index and cardiovascular disease risk, aligning with prior mechanistic research. When BMI increases, more fat cells grow and enlarge. Adipose tissue secretes various cytokines and hormones, such as leptin and adiponectin, which affect the insulin signaling pathway, leading to insulin resistance. In an insulin-resistant state, the liver becomes less sensitive to insulin, increasing gluconeogenesis and glycogenolysis, thus raising blood glucose levels. Obesity caused by elevated BMI can trigger chronic inflammatory responses. Immune cells in adipose tissue, such as macrophages, release large amounts of inflammatory factors, including tumor necrosis factor-α and interleukin-6. These inflammatory factors damage vascular endothelial cells, increase their permeability, promote lipid deposition in the vessel wall, and accelerate the formation of atherosclerotic plaques (25). However, in the relationship between high BMI and atrial fibrillation (AF), BMI induces structural and electrophysiological remodeling of the left atrium, exacerbates myocardial fibrosis, and triggers systemic inflammation (26, 27). At the same time, inflammatory responses can disrupt the balance of coagulation and fibrinolysis systems, placing the body in a pro-coagulant state, making it more prone to thrombosis and increasing the risk of cardiovascular events. People with high BMI often have abnormal lipid metabolism, characterized by elevated triglycerides and decreased high-density lipoprotein cholesterol. An increase in BMI promotes the accumulation of lipids within fat cells and affects the liver's synthesis, transport, and metabolism of lipids. The TyG index includes triglyceride and blood glucose levels. When a high BMI leads to lipid metabolic disorders, triglyceride levels rise, directly impacting the TyG index. Abnormal lipid metabolism is a significant risk factor for CVD, and insulin resistance is also an important factor in the development of CVD. These metabolic disorders severely promote the formation and progression of atherosclerotic plaques, particularly through impaired high-density lipoprotein function, which damages the vascular endothelial protective mechanism (28, 29). The established pathophysiological link between dyslipidemia and cardiac metabolic disorders——especially type 2 diabetes and metabolic syndrome——further confirms the clinical validity of the TyG index as a biomarker for cardiovascular risk stratification (30, 31).

The relationship between TyG and CVD is influenced by multiple factors. For example, elevated blood pressure can lead to endothelial dysfunction, increasing the risk of CVD, and may interact with TyG to affect the risk of CVD. Systemic inflammation, as indicated by markers such as C-reactive protein, may also play a role in the development of CVD, and its relationship with TyG and CVD is worth further exploration. In addition to these mediating factors, genetic factors, lifestyle (such as diet, exercise, smoking, and alcohol consumption), other metabolic indicators (such as cholesterol levels and uric acid levels), and personal factors are also involved. For instance, genetic factors may influence an individual's susceptibility to insulin resistance, thereby affecting the relationship between TyG and CVD; unhealthy lifestyles, such as high-fat and high-sugar diets and lack of exercise, not only lead to increased TyG levels and higher CVD risk but can also exacerbate the link between TyG and CVD by intensifying insulin resistance and inflammatory responses. These factors interact, collectively influencing the relationship between TyG and CVD, making their connection extremely complex. Future comprehensive studies incorporating these factors will help us gain a deeper understanding of their underlying mechanisms.

### 4.1 Strengths and limitations

So far, this is the first time two large-scale surveys have been combined to study the association between TyG and CVD, breaking the limitations of previous single-race studies. Additionally, by conducting a mediation analysis of BMI in a large-scale sample survey, this study not only advances the existing understanding of the correlation between the TyG index and the pathogenesis of cardiovascular disease but also provides new insights into its use as a cost-effective screening tool for early disease identification in primary care settings. This offers a fresh perspective on future research into the underlying mechanisms of cardiovascular disease risk.

While leveraging substantial sample size with population representativeness, this investigation bears inherent methodological constraints. Primarily, the observational study design precludes definitive causal inference between TyG index and cardiovascular outcomes. Secondly, the study lacks detailed information on diet and exercise, which may affect the comprehensive assessment of lifestyle factors. Additionally, the inclusion and exclusion criteria for both NHANES and CHARLS may limit the representation of certain populations, particularly in terms of racial and regional diversity. Methodological limitations arising from incomplete datasets precluded full adjustment for confounding variables, including hereditary influences and therapeutic regimens. Finally, in the logistic regression main effect model used in this study, interaction terms are included as factors. The conclusion about whether there is an interaction depends on whether the interaction term is meaningful. The Logistic interaction term only reflects statistical data interactions, and can only be interpreted as having a multiplicative interaction effect; therefore, interaction *P*-values should be interpreted with caution. Future research should focus on conducting longitudinal cohort studies to establish the causal relationship between the TyG index and CVD risk and examine its dynamic changes across different populations. Moreover, gene expression analysis, metabolomics, and other advanced methods should be used to investigate the molecular mechanisms underlying insulin resistance, providing a theoretical basis for targeted prevention strategies. Interventional studies should explore the effects of insulin resistance interventions, such as lifestyle changes and pharmacotherapy, on reducing cardiovascular disease risk. Lastly, future research should also investigate racial and regional differences to better understand the global applicability of the TyG index.

## 5 Conclusion

This investigation delineated significant associations among TyG index, BMI, and cardiovascular risk through dual population-based cohorts. Mechanistic analyses identified BMI as a pivotal mediator in the TyG index-cardiovascular disease pathway. The results showed that the increase of BMI was associated with insulin resistance, and synergistically with the increase of TyG index, which disrupted glucose-lipid homeostasis and thus affected the pathogenesis of cardiovascular disease.

## Data Availability

The datasets presented in this study can be found in online repositories. The names of the repository/repositories and accession number(s) can be found in the article/Supplementary Material.
